# The temporal order of fluctuations in atopic disease symptoms and attention-deficit/hyperactivity disorder symptoms: a time-series study in ADHD patients

**DOI:** 10.1007/s00787-019-01336-2

**Published:** 2019-04-24

**Authors:** Jurjen van der Schans, Qi Cao, Elisabeth H. Bos, G. Ingrid J. G. Rours, Pieter J. Hoekstra, Eelko Hak, Tjalling W. de Vries

**Affiliations:** 1 PharmacoTherapy, Epidemiology and Economics, Groningen Research Institute of Pharmacy, University of Groningen, Antonius Deusinglaan 1, 9713 AV Groningen, The Netherlands; 2 Department of Developmental Psychology, Faculty of Behavioural and Social Sciences, University of Groningen, Grote Kruisstraat 2/1, 9712 TS Groningen, The Netherlands; 3 Medical Center for Quality of Life, Kinderplein, Metroplein 88, 3083 BB Rotterdam, The Netherlands; 4 Department of Child and Adolescent Psychiatry, University Medical Center Groningen, University of Groningen, Hanzeplein 1, 9713 GZ Groningen, The Netherlands; 5 Department of Epidemiology, University Medical Center Groningen, Hanzeplein 1, 9713 GZ Groningen, The Netherlands; 6 Department of Pediatrics, Medical Center Leeuwarden, Henri Dunantweg 2, 8934 AD Leeuwarden, The Netherlands

**Keywords:** Attention-deficit/hyperactivity disorder, Atopy, Pediatrics, Daily diary study

## Abstract

In a recent meta-analysis, we found that atopic diseases, like asthma and allergic rhinitis, occur more frequently prior to the onset of attention-deficit/hyperactivity disorder (ADHD). Our aim was to determine the temporal order of the association between daily fluctuations in atopic disease symptoms and in ADHD symptoms in individual participants. In this observational study among 21 participants, age 7–16 years, we performed a replicated time-series analysis of symptom fluctuations in asthma and/or allergic rhinitis and ADHD. Data were collected through parents who filled in a daily online questionnaire during up to 50 days. In each individual, we investigated the temporal order of fluctuations in atopic disease symptoms and ADHD symptoms using a vector autoregressive (VAR) model while using sleep problems and medication use as covariates. For 16 out of 21 participants, we constructed a VAR model. For a majority of the participants, significant associations were detected between atopic disease symptoms and ADHD symptoms. The results were heterogeneous; the direction, sign, and timing of the relationship between ADHD, atopy, sleep problems, and medication use varied between individuals. This study provides additional evidence that the symptom expression of atopy and ADHD are related. However, the connection between both diseases in children is found to be heterogeneous within our study population.

## Introduction

Attention-deficit/hyperactivity disorder (ADHD) is a common neurobehavioral disorder with onset of symptoms before the age of 12 years [1], characterized by symptoms of inattention and hyperactivity/impulsivity that often lead to social impairments. ADHD is a multifactorial disorder associated with both genetic and environmental factors, possibly including nutrition [2].

ADHD and atopic diseases co-occur more often than would be expected on the basis of chance [3]. Studies on the association between atopic diseases and ADHD have shown conflicting results in the strength of an association. However, almost all detected an increased risk in either the presence of an atopic disease in those with ADHD or vice versa an increased risk of the development of ADHD in those with an atopic disease [4]. Large cohorts with a few measurement waves are only informative to detect general patterns, and are not suitable for the detection of dynamic relationships between symptoms on a patient level [5].

In view of the frequent co-occurrence of ADHD and atopic diseases, it is important to investigate the possibility of an underlying common causal pathway. Daily fluctuations in symptoms are a hallmark of both atopic diseases and ADHD. The expression of symptoms in both atopy and ADHD is among others dependent on treatment efficiency, compliance to treatment, and external factors, which may cause these symptom fluctuations in both diseases. By studying these fluctuations over time within a series of individual ADHD cases and examining the dynamic association between atopic symptom fluctuations and ADHD symptom fluctuations, we aimed to determine the temporal order of the association between symptoms of atopic diseases and symptoms of ADHD.

## Methods

### Study design

We performed a replicated time-series analysis of asthma and allergic rhinitis symptom fluctuations and ADHD symptom fluctuations in individual participants. The time-series data were collected by means of an online diary study. For a period of 50 days, one of the parents was asked to fill out daily questionnaires; to be done at a specific time, preferably the end of the day between 8 and 9 pm. The questionnaire was applied as a mobile phone application and was used to assess the asthma and allergic rhinitis symptoms, and the ADHD symptoms. One and the same parent were asked to fill out the baseline questionnaire and the daily questionnaires to prevent reporting bias.

### Study population

The study population consisted of 21 children in the age of 7–16 years in whom the diagnosis ADHD was established by a child psychiatrist, a child psychologist, or a pediatrician. Asthma or allergic rhinitis was diagnosed by a physician. Children were excluded if they were diagnosed with any chronic disease other than atopic diseases or ADHD. Parents could not participate if they were not fluent in the Dutch language or were, otherwise, unable to fulfill the study procedures or had no access to the Internet. Children and their parents were recruited by their pharmacists through the IADB.nl prescription database and the patient database of a medical center in Rotterdam. Participants were asked to continue care as usual throughout the study. Our local medical ethical committee (METC UMCG) had waived the ethical approval for this observational study design according to the declaration of Helsinki. All participants were asked to fill in an informed consent form before participation of the study.

### Measures

At baseline, for each participant, age, sex, and the ratings of the ADHD symptoms [26-item scale Swanson, Nolan, and Pelham Questionnaire (SNAP-26)], atopy [Control of Allergic Rhinitis and Asthma Test (CARAT)], and sleep problems [Children’s Sleep Habits Questionnaire (CSHQ)] as rated by one of the parents in the week before the initiation of the study, were determined to describe the sample characteristics.

To assess the severity of ADHD symptoms during the diary period, we used the SNAP IV questionnaire, which is a frequently used, validated tool in ADHD studies that is based on the 18 DSM-IV ADHD items [6, 7]. The combined score of inattention and hyperactivity/impulsivity subscale scores was used as rating of ADHD symptoms (18 questions; total score range, 0–54). To assess the asthma and allergic rhinitis symptom severity the adjusted CARAT questionnaire was used (7 questions; total score range, 0–21). The CARAT questionnaire is a validated and consistent way to measure asthma and allergic rhinitis symptoms [8]. Currently, the Adapt Asthma application, which is based on the CARAT, is already in use to monitor asthma and rhinitis symptoms [9]. The questions and answer options of the SNAP and the CARAT questionnaire were adapted for daily measurement by changing the phrasing and time periods of the original questions were referring to. In total, parents needed to answer seven questions for atopy and eighteen questions for ADHD daily, with different response categories per question.

### Covariates

Co-variables like age, gender, sleep problems, and medication use were determined during inclusion and as part of the daily measurement. To address the possible involvement of sleep problems, as suggested by the previous research into atopic eczema and ADHD [10, 11], in the fluctuations in ADHD symptoms and atopic symptoms, we added eight questions concerning assessment of daytime sleepiness selected from the CSHQ questionnaire (8 questions; total score range, 7–31) to the daily diary. Higher scores reflect worse sleep. The questions and answer options of the CSHQ questionnaire were adapted for daily measurement during daytime by changing the phrasing and time periods the original questions were referring to. In addition, items on medication use of both atopic and ADHD medication were added to the daily diary (‘How often did your child take a higher dosage of its regular medication due to allergic rhinitis and/or asthma?’; ‘How often did your child take a higher dosage of its regular medication due to ADHD symptoms?’; total score range, 0–3) to account for the possible effects of fluctuations in medication use on symptom expression in the models. Sleep problems and medication use were added as a covariate instead of a confounder, because of the association reported between ADHD and atopic diseases independent of sleep and medication [4].

### Statistical analyses

A vector autoregressive (VAR) model was used to investigate the dynamic associations between changes in symptoms in each individual participant as well as the possible involvement of covariates in this association [12]. An important feature of the VAR model is the possibility to examine the temporal dynamics between multiple time-series, which allows studying the temporal order of the association between atopy and ADHD symptoms accounting for potential bidirectional effects and effects of time-varying covariates like sleep problems and medication use [12, 13]. Another important feature of the VAR technique is the ability to separate the dynamic longitudinal part from the simultaneous part of the associations between the variables and to correct for potential feedback effects [12].

A VAR model consists of a set of regression equations, in which each of the variables is regressed on its own lagged values (autocorrelation) as well as the lagged values of the other variables (cross-lagged associations) [19]. We build VAR models with two endogenous variables: atopy symptoms and ADHD symptoms. The variable sleep problems were added to the primary model as an extra endogenous variable, because sleep can influence and can be influenced by atopy and ADHD symptoms. Medication use was added to the model as an endogenous variable if there was variation in medication use during the follow-up period. We first investigated the optimal lag number to be used in each of the models (a lag = 1 day), according to a comparison of the final prediction error of each lag model. Since VAR models assume stationarity, we subsequently compared four different types of VAR models (constant, trend, both, and none) to determine if an additional trend or intercept term was needed in the model. Finally, we selected the optimal model with the Akaike Information Criterion (AIC) based on the smallest value of AIC, a measure of relative quality of the fitted model.

To assess the temporal order of the association between atopy and ADHD symptoms, and the possible involvement of sleep problems therein, we determined the independent cross-lagged associations between each variable. In addition, we assessed the contemporaneous correlations between symptoms of atopy, ADHD, and sleep problems per participant to determine the simultaneous associations between (or co-occurrence of) the expression of symptoms. These can be computed from the residuals of the VAR model [19]. A *p* value ≤ 0.05 was considered to be statistically significant. Per individual model, multiple diagnostic tests were performed to test for stability and homoscedasticity of the model [14]. For six out of the participants, variables were log-transformed for either the score of atopy (participants 10, 11, 13, and 16) or for both atopy and sleep (participants 2 and 3), because the score itself is not normally distributed as observed by the histogram. Models were fitted in R (R Foundation for Statistical Computing, software version 3.4.0, Vienna, Austria) using the package vars. *p* values < 0.05 were considered to denote statistically significant associations and *p* values < 0.10 were considered to denote the trend of associations.

Simulation studies have shown that, for VAR modeling, a minimum of 30 time points is needed, although larger numbers of observations yield more reliable results [14]. We chose for a time-series length of 50 measurement points taking into account the possibility of missing data. Missing values on the daily sum scores of ADHD, allergy, and sleep were dealt with using multiple imputations on the individual level [15]. Considering the percentage of the missing measurements per participant, we obtained five imputed data sets for participants 1, 2, 3, 4, 9, 10, 11, 13, and 16, ten imputed data sets for participants 5, 7, 15, 17, 18, and 21, and 15 imputed data sets for participants 19. The imputed disease ratings were then computed by taking the average scores generated in each imputation. Subsequently, ADHD ratings were calculated by summing up the separate inattentive score and hyperactive score.

## Results

Five of the twenty-one participants (#6, #8, #12, #14, and #20) were excluded from analyses, because not enough observations (*n* < 30) were completed during the data collection. A description of the characteristics of our study sample is presented in Table 1. All participants were between 7 and 16 years of age at the start of the study period. The majority (61.9%) were male. Per participant, the baseline ratings of ADHD symptoms (SNAP-26), atopy (CARAT), and sleep (CSHQ) are shown. Within our study sample, nine out of the twenty-one participants (42.9%) were diagnosed with both asthma and allergic rhinitis.

**Table 1:** Characteristics of the study group (*n* = 21)

Characteristics	Mean (SD)	Participants																				
		1	2	3	4	5	6	7	8	9	10	11	12	13	14	15	16	17	18	19	20	21
Completed observations		39	48	46	49	30	5	30	22	43	44	42	21	50	21	37	43	32	40	32	4	42
Gender (female), *n*	8 (38.1%)	♀	♂	♂	♂	♂	♂	♀	♀	♂	♂	♂	♀	♂	♂	♂	♂	♀	♀	♂	♀	♀
Age	11.3 (2.3)	12	11	12	7	11	16	13	10	9	12	8	12	11	12	11	15	10	14	13	12	7
ADHD diagnosis		+	+	+	+	+	+	+	+	+	+	+	+	+	+	+	+	+	+	+	+	+
Asthma diagnosis		+	−	−	+	+	+	−	−	+	−	+	+	−	−	+	+	+	−	−	−	+
Allergic rhinitis diagnosis		+	+	+	+	+	+	+	+	+	+	+	+	+	+	−	+	−	+	+	+	+
SNAP-26 score																						
Total	34.6 (10.6)	60	37	12	40	35	39	31	32	17	18	39	34	30	48	36	40	31	41	43	36	27
Inattention	18.7 (4.7)	19	17	11	18	17	23	20	15	9	17	21	17	14	27	14	20	27	19	26	21	20
Hyperactivity/impulsivity	11.0 (5.7)	18	11	1	20	15	8	9	15	7	0	14	17	13	18	16	11	1	10	9	12	7
CARAT score	10.1 (5.6)	7	6	7	12	17	4	11	4	8	8	7	10	12	28	15	11	17	11	8	6	4
CSHQ score	47.8 (8.1)	60	52	42	45	53	54	56	37	37	38	37	49	51	68	47	53	40	46	48	45	45
Overall sleep grade (0–10)	6.8 (1.8)	4	8	8	7	5	3	5	8	9	8	9	6	7	5	4	8	9	7	7	7	8

*SD* standard deviation, *n* sample size, *ADHD* attention-deficit/hyperactivity disorder, *SNAP* Swanson Nolan and Pelham questionnaire, *CARAT* control of allergic rhinitis and asthma test, *CSHQ* children’s sleep habits questionnaire

♀ girl, ♂ boy, + yes, − no

### Temporal order of the relationship between atopy, ADHD, and sleep problems

In all models, an optimal lag of 1 day was selected to be optimal. Both the temporal direction as well as the sign (positive or negative) and size of the effect varied between the participants. Five out of the 16 participants showed a significant (*p* < 0.05) cross-lagged association between some of the variables (Table 2). In participant 1, an increase in expression of atopic symptoms preceded a worsening in ADHD symptoms (*B* = 3.59; *p* = 0.004) and sleep problems (*B* = 0.71; *p* = 0.023), while increases in sleep problems were followed by decreases in ADHD symptoms (*B* = − 1.51; *p* = 0.013). In participant 10, an increase in ADHD symptoms was followed by an increase in symptoms of atopy (*B* = 0.12; *p* = 0.007). In contrast to the other participants, in participant 11, a negative association was detected in which a decrease in atopic symptoms (*B* = − 1.25; *p* = 0.048) was followed by an increase in ADHD symptoms. Participant 15 showed a worsening of sleep, preceded by an increase in ADHD symptoms (*B* = 0.13; *p* = 0.045). In participant 18, sleep problems and atopic symptoms were associated in both directions, i.e., an increase in sleep problems preceded an increase in atopic symptoms (*B* = 0.33; *p* = 0.032), while an increase in atopic symptoms preceded a decrease in sleep problems (*B* = − 0.48; *p* = 0.004).

**Table 2:** Significant and trend 1-day cross-lagged associations

Participant ID	Atopy → ADHD		ADHD → atopy		Sleep → ADHD		ADHD → sleep		Sleep → atopy		Atopy → sleep	
	*B*	*p* value	*B*	*p* value	*B*	*p* value	*B*	*p* value	B	*p* value	*B*	*p* value
1	3.59	0.004			− 1.51	0.013					0.71	0.023
2^a^					− 9.27	0.075						
3^a^												
4			− 0.13	0.056								
5												
7												
9	0.40	0.052	0.22	0.051								
10^a^			0.12	0.007								
11^a^	− 1.25	0.048							− 0.07	0.058		
13^a^												
15			0.079	0.096			0.13	0.045			0.31	0.084
16												
17												
18^a^									0.33	0.032	− 0.48	0.004
19							0.30	0.097				
21^a^	− 0.42	0.071							− 0.35	0.052		

→ dynamic effect, *atopy* sum score of atopic symptoms, *ADHD* sum score of attention-deficit/hyperactivity disorder symptoms, *sleep* sum score of sleep problems, *B* unstandardized regression coefficient

^a^Models are adjusted for ADHD medication use (participants 2, 3, 10, 11, 13, and 18) and allergic medication use (participants 10, 13, and 21)

### Contemporaneous associations

In addition to the dynamic (cross-lagged) associations between symptoms of atopy, ADHD, and sleep problems, we also addressed the co-occurrence of these symptoms. Table 3 shows an overview of the contemporaneous expression of symptoms of atopy, ADHD, and sleep problems. Atopy and ADHD, sleep problems and ADHD, and sleep problems and atopy were contemporaneously associated with a number of participants. In 4 out of 16 participants, the contemporaneous association between atopy and ADHD was significant. The sign of this association was positive in 3 out of 4 of these participants. Also most of the contemporaneous associations between sleep problems and atopy or ADHD symptoms had a positive sign.

**Table 3:** Contemporaneous significant and trend associations between symptoms of atopy, ADHD, and sleep problems

Participant ID	Atopy and ADHD		Sleep and ADHD		Sleep and atopy	
	*r*	*p* value	*r*	*p* value	*r*	*p* value
1						
2^a^	− 0.29	0.093	− 0.24	0.045	0.40	0.004
3^a^						
4			0.26	0.067		
5					− 0.70	< 0.001
7	0.28	0.080			0.27	0.093
9						
10^a^	0.31	0.032	− 0.45	0.001		
11^a^						
13^a^						
15						
16	0.30	0.040	0.25	0.089		
17	− 0.43	0.003				
18^a^	0.27	0.056				
19						
21^a^	0.66	< 0.001	0.34	0.014	0.50	< 0.001

*ADHD* attention-deficit/hyperactivity disorder, *r* correlation coefficient

^a^Models are adjusted for ADHD medication use (participants 2, 3, 10, 11, 13, and 18) and allergic medication use (participants 10, 13, and 21)

### Associations between medication use and the symptom variables/sleep problems

In seven participants, the medication use related to ADHD (participants 2, 3, 10, 11, 13, and 18) and to allergy (participants 10, 13, and 21) varied during the study period. For this reason, these variables were included in the VAR models of these participants as an additional endogenous parameter. A number of cross-lagged associations between medication use and the other variables were present: Participant 10 showed a significant cross-lagged association between sleep problems (*B* = 0.14; *p* = 0.048) and ADHD medication: an increase in sleep problems was followed by an increase in ADHD medication. In participant 21, significant cross-lagged associations between allergic-related medication on one hand, and ADHD (*B* = 4.98; *p* = 0.009), sleep problems (*B* = − 0.04; *p* = 0.012), and atopy (*B* = 0.06; *p* = 0.006) on the other hand were detected.

When evaluating the contemporaneous associations between medication use and the symptom expression of the different diseases, we detected a negative association between ADHD medication and atopy (*r* = − 0.29; *p* = 0.042) in participant 2 and a negative association between ADHD medication and sleep problems (*r* = − 0.36; *p* = 0.010) in participant 2 and (*r* = − 0.29; *p* = 0.044) participant 10. Furthermore, we observed a positive association between allergic medication and ADHD (*r* = 0.34; *p* = 0.013) and between allergic medication and atopy (*r* = 0.66; *p* < 0.001) in participant 21.

In participants 3, 11, 13, and 18, no association was observed between medication use and either sleep, ADHD, and/or atopy.

### Differentiating inattention and hyperactivity/impulsivity

When stratifying the ADHD sum score into separate scores of inattention and hyperactivity/impulsivity, similar results were observed. A notable difference was that the cross-lagged association between sleep problems and ADHD symptoms in participant 2 appeared to be mainly accounted for by hyperactivity/impulsivity symptoms. The same was true for the cross-lagged association between ADHD and atopic symptoms in participant 4 and between ADHD and sleep problems in participant 15, which were also accounted for by hyperactivity/impulsivity symptoms. The opposite was true for the cross-lagged association between ADHD symptoms and atopic symptoms in participant 10, which was accounted for by symptoms of inattention.

A separate cross-lagged association appeared in participant 3 for between inattention and sleep problems (*B* = 0.02; *p* = 0.072) and in participant 16 between inattention and atopic symptoms (*B* = 0.07; *p* = 0.031).

As regards the contemporaneous associations between the different symptoms, also similar results were observed as in the original analyses. The contemporaneous association between atopy and ADHD, and sleep problems and ADHD in participant 10, atopy and ADHD in participant 16, and sleep problems and ADHD in participant 21 were accounted for by symptoms of inattention. In comparison with Table 3, new significant associations were found between atopy and inattention (*r* = 0.40; *p* = 0.005), and sleep problems and inattention (*r* = 0.37; *p* = 0.010) in participant 4, sleep problems and inattention (*r* = 0.46; *p* = 0.002) in participant 7, and sleep problems and hyperactivity/impulsivity (*r* = − 0.37; *p* = 0.010) in participant 9.

## Discussion

To our knowledge, this is the first study using time-series analysis to examine the temporal order of an association between atopic disease symptoms and ADHD symptoms. For a majority of the participants, significant associations were detected between atopic disease symptoms and ADHD symptoms. However, the results were heterogeneous, with varying direction, sign, and timing of the relationship between ADHD, atopy, sleep problems, and medication use between participants. In other words, the observed associations were not consistent across the participants. Regarding ADHD symptoms as outcome, our analysis showed a relationship between symptoms of allergy or sleep problems and ADHD symptoms in some of the participants. There was also negative cross-lagged association present between sleep problems and ADHD. The cross-lagged association between atopy and ADHD differed in direction between the participants. Regarding atopic symptoms as the outcome, only one participant showed a significant cross-lagged association between ADHD and allergy symptoms. When assessing the more direct contemporaneous relationships, our analyses showed an association in many of the participants between ADHD symptoms (inattention, hyperactivity/impulsivity, or combined) or ADHD medication use, allergy, and/or sleep problems. The associations differed in both sign and strength.

Based on different group-based observational studies, the association between atopy and ADHD is considered weak-to-moderate [3, 16]. Although the association between symptom patterns in some participants does correspond with these between-subject results, in other participants, the relationship between ADHD and atopy was absent or even reversed from what would be expected based on the literature of between-subject results. This might be explained by the fact that both atopic disease and ADHD are complex and heterogeneous diseases with regards to etiology, symptom expression, and treatment [17, 18]. As a second reason for the heterogeneity of our results, proxy reporting by parents may be less suitable for assessing sleep problems and symptoms of allergy. Another reason may be that our series were rather short, which has limited our power. Future studies should preferably collect longer series. In general, the heterogeneity of our study results may explain why the effect size of the association between atopy and ADHD in group-based studies is often weak. In a heterogeneous population, in which the association between atopy and ADHD is not present in every individual, the group-based association between the two diseases can be diluted, while the association between the diseases can still be significant at an individual level. This is illustrated by our results in which the cross-lagged association between atopy and subsequent ADHD symptoms is reversed in participant 1 compared to participant 11, while both show a significant association between the symptoms. A similar pattern is evident when comparing the between-subjects correlation and the within-subject correlation, representing the correlation over individuals and over time, respectively. Participants 5 and 7 both scored relatively high on the baseline score of atopy and sleep problems compared to the other participants, while at an individual level over time atopy and sleep problems showed a negative contemporaneous correlation in participant 5 and a positive contemporaneous correlation in participant 7.

### Clinical relevance

A recent study showed that treatment of allergic rhinitis decreased ADHD symptom severity significantly compared with healthy controls and children with pure ADHD [19]. In our study, in only one participant, increases in allergic symptoms were followed by subsequent increases in ADHD symptoms, suggesting that a specific treatment of the atopic symptoms might, perhaps, improve the symptoms of ADHD in this individual. On the contrary, treatment of ADHD might be beneficial for the symptoms of allergy of participant 10 and participant 16, because increases in ADHD symptoms were followed by increases in atopic symptoms in these participants. Participants 4 (inattention), 10, 16, and 21 showed a contemporaneous positive association between allergic and ADHD symptoms, in which an improvement of atopic symptoms could potentially benefit the participant by also improving the ADHD symptom expression, and vice versa. Because of a direct association between ADHD symptoms and sleep problems in participants 3 (inattention) and 15, and a contemporaneous positive association between ADHD and sleep problems in participants 4 (inattention), 7 (inattention), and 21, these participants might benefit from improving their sleep for amelioration of their ADHD symptoms. Adding to the complexity of the different associations between ADHD, atopy, sleep problems, and the subsequent possible clinical implications is the use of medication. In, for example, participant 21, lowering of allergic medication could possibly benefit the participant in her ADHD symptoms. However, a direct positive association still exists between symptoms of allergy and ADHD when controlling for medication use, which would suggest a contradictory clinical implication of an increase in treatment of allergy and/or ADHD to improve symptoms of both disorders.

Taking into account the group-based cross-sectional and prospective association between atopy and ADHD in the literature [3], it might be expected that this study would have shown a more consistent direct or lagged effect between ADHD and atopy. Because no such consistent relationship was established in this study, it is possible that the association between atopy and ADHD partly exists because of an indirect mechanism, like genetic predisposition, or that the temporal association of cause and effect is stretched over a longer period of time. Multiple pathophysiological hypotheses have been raised to explain the possible comorbidity between atopic diseases and ADHD [20]. Among others, chronic stress and chronic inflammation have been raised as possible connections between atopy and ADHD. Because of the limited study period, it was not possible to measure these possible effects. While a general group-based approach of treating atopy to improve ADHD symptoms, and vice versa, will not necessarily benefit the whole group, individualized assessment of interdisciplinary treatment of both diseases could benefit individual participants.

### Strengths and limitations

Our findings should be interpreted in the light of the strengths and limitations. Examining symptom fluctuations in allergy, ADHD, and sleep problems on a daily basis allowed us to observe the concurrent as well as the temporal relationships between the different variables. A limitation was the small sample size of the study, which limits the generalizability of the results. However, due to the multiple repeated measurement points per participant, it was possible to examine each individual participant in detail and to explore the possible temporal order of the association between atopy and ADHD. Combining both study types, with repeated measurements of symptom expression of both atopy and ADHD in a large cohort, would benefit the study of the association between both diseases.

Another limitation has been that the participants were followed daily for a maximum of 50 days, but the effects of the symptom expression of one of the diseases could accumulate over time to have a larger and more significant effect on the long term [21, 22]. For example, weekly measurements over a longer period of time could reveal a more consistent association between the symptom expressions of both diseases. In addition, adding atopic eczema, food allergies, and a more comprehensive assessment of sleep problems would be recommendable for future studies. The role of age of onset and disease duration in examining the association between symptoms of ADHD, atopy, and sleep problems may also be considered in future studies. We did not add more variables to our daily questionnaire to avoid loss to follow-up of our participants.

Time-series analysis is a potentially useful method to assess the temporal order of the association between atopy and ADHD. This study provides additional evidence that the symptom expression of atopy and ADHD is related. However, the connection between symptom expressions of both diseases in children is heterogeneous, and differs in sign, direction, strength, and timing in our study population. To enable improvement in the care of patients, personalized treatment needs of children with both atopic disease and ADHD should be assessed at an individual basis.
